# Local Melatoninergic System as the Protector of Skin Integrity

**DOI:** 10.3390/ijms151017705

**Published:** 2014-09-30

**Authors:** Andrzej T. Slominski, Konrad Kleszczyński, Igor Semak, Zorica Janjetovic, Michał A. Żmijewski, Tae-Kang Kim, Radomir M. Slominski, Russel J. Reiter, Tobias W. Fischer

**Affiliations:** 1 Department of Pathology and Laboratory Medicine, Cancer Research Building, University of Tennessee HSC, 930 Madison Avenue, Memphis, TN 38163, USA; E-Mails: zjanjeto@uthsc.edu (Z.J.); tkim10@uthsc.edu (T.-K.K.); radomir.slominski@gmail.com (R.M.S.); 2 Department of Medicine, Division of Rheumatology, University of Tennessee HSC, 930 Madison Avenue, Memphis, TN 38163, USA; 3 Department of Dermatology, Allergology and Venerology, University of Lübeck, Ratzeburger Allee 160, 23538 Lübeck, Germany; E-Mails: Konrad.Kleszczynski@uksh.de (K.K.); Tobias.Fischer@uksh.de (T.W.F.); 4 Department of Biochemistry, Belarusian State University, Minsk 220030, Belarus; E-Mail: semak@bsu.by; 5 Department of Histology, Medical University of Gdańsk, Gdańsk 80-211, Poland; E-Mail: mzmijewski@gumed.edu.pl; 6 Department of Cellular and Structural Biology, UT Health Science Center, San Antonio, TX 78229, USA; E-Mail: REITER@uthscsa.edu

**Keywords:** melatonin, keratinocytes, AFMK, 6-hydroxymelatonin, human full-thickness skin, ultraviolet radiation, oxidative stress

## Abstract

The human skin is not only a target for the protective actions of melatonin, but also a site of melatonin synthesis and metabolism, suggesting an important role for a local melatoninergic system in protection against ultraviolet radiation (UVR) induced damages. While melatonin exerts many effects on cell physiology and tissue homeostasis via membrane bound melatonin receptors, the strong protective effects of melatonin against the UVR-induced skin damage including DNA repair/protection seen at its high (pharmocological) concentrations indicate that these are mainly mediated through receptor-independent mechanisms or perhaps through activation of putative melatonin nuclear receptors. The destructive effects of the UVR are significantly counteracted or modulated by melatonin in the context of a complex intracutaneous melatoninergic anti-oxidative system with UVR-enhanced or UVR-independent melatonin metabolites. Therefore, endogenous intracutaneous melatonin production, together with topically-applied exogenous melatonin or metabolites would be expected to represent one of the most potent anti-oxidative defense systems against the UV-induced damage to the skin. In summary, we propose that melatonin can be exploited therapeutically as a protective agent or as a survival factor with anti-genotoxic properties or as a “guardian” of the genome and cellular integrity with clinical applications in UVR-induced pathology that includes carcinogenesis and skin aging.

## 1. Synthesis and Metabolism of Melatonin in a “Nutshell”

### 1.1. Overview of Melatonin Synthesis

The structure of melatonin (*N*-acetyl-5-methoxytrypamine) was defined over 50 years ago by Aaron B. Lerner after its isolation from the bovine pineal gland [1]. The synthesis of melatonin is a four step process that begins with the amino acid l-tryptophan and ends with the production of melatonin [2]. The first stage is the hydroxylation of the substrate l-tryptophan to 5-hydroxytryptophan by tryptophan hydroxylase (TPH) [3,4,5]. There are two isoforms of tryptophan hydroxylase identified as TPH1 and TPH2. TPH1 is expressed in many peripheral tissues [6,7,8] including the skin [9,10], while TPH2 is expressed in neuronal cells and is the predominant isoform in the central nervous system [6,11]. The second stage of melatonin synthesis involves decarboxylation of 5-hydroxytryptophan to serotonin by amino acid decarboxylase (AA) [12]. In the third step, arylalkylamine *N*-acetyltransferase (AANAT) acetylates serotonin to *N*-acetylserotonin [13,14,15]. The final step is the conversion of *N*-acetylserotonin to melatonin by hydroxyindole-*O*-methyltransferase (HIOMT) [16]; the latter enzyme is sometime identified as *N*-acetylserotonin methyltransferase.

There are three rate limiting steps in melatonin synthesis that are mediated by TPH, AANAT, and HIOMT, with tryptophan hydroxylation representing a common rate limiting enzyme for both serotonin and melatonin [17]. Although AANAT is considered as the rate limiting step in melatonin synthesis [14], Liu and Borjigin have shown that rats with the H2BY mutation in the AANAT, which drastically reduces enzyme activities, have normal melatonin levels in their pineal gland [18]. These findings are consistent with observations in mammalian skin showing that serotonin is acetylated in peripheral tissue by an alternative enzyme to AANAT, most likely arylamine *N*-acetyltransferase [9,10,19,20], including in the C57BL6 mouse [10,21]. The above information indicates that only HIOMT should be considered a rate limiting enzyme in melatonin synthesis [17].

### 1.2. Melatoninergic System in the Skin

**Figure 1:**
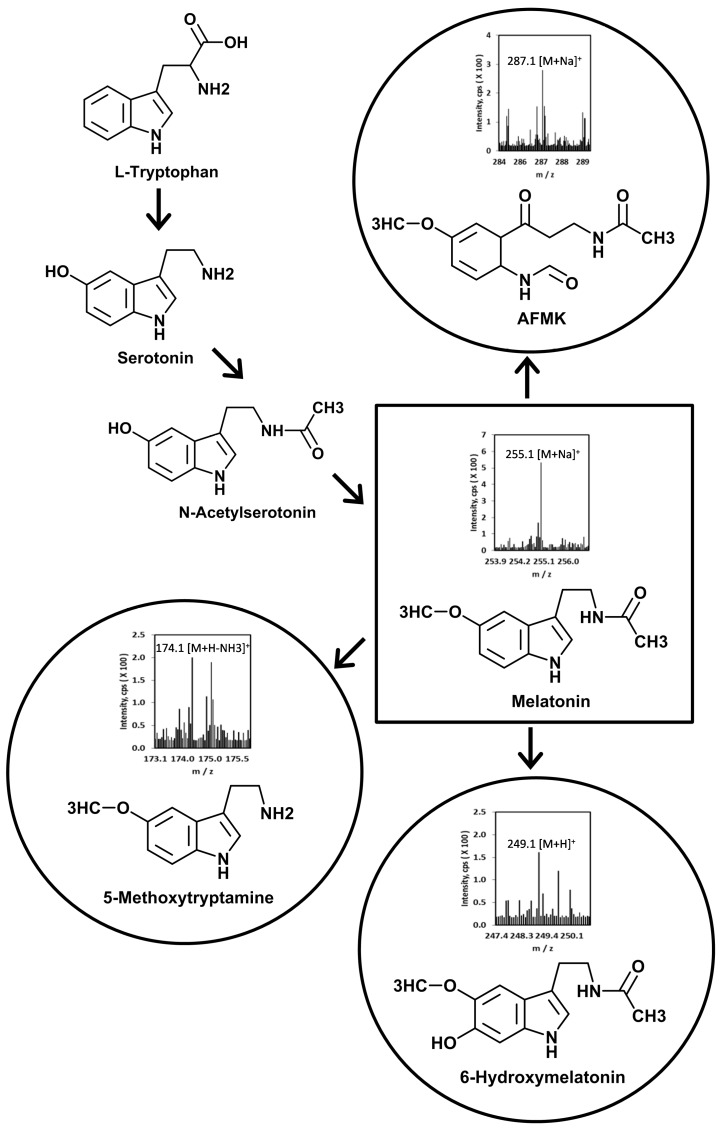
Scheme of cutaneous melatonin synthesis and metabolism. Mass spectra of melatonin metabolites are from separated fraction with retention times of the corresponding standards [31].

The human skin and skin cells express all steps of melatonin synthesis, including the rate limiting enzymes TPH1, AANAT, and HIOMT [9,22,23,24,25,26,27,28] (Figure 1). In addition, rodent skin has the capability of producing melatonin using classical and alternative (AANAT independent) pathways from serotonin [9,10,19,20,23,29]. Furthermore, retinal pigment epithelium shares the same property [30].

**Figure 2:**
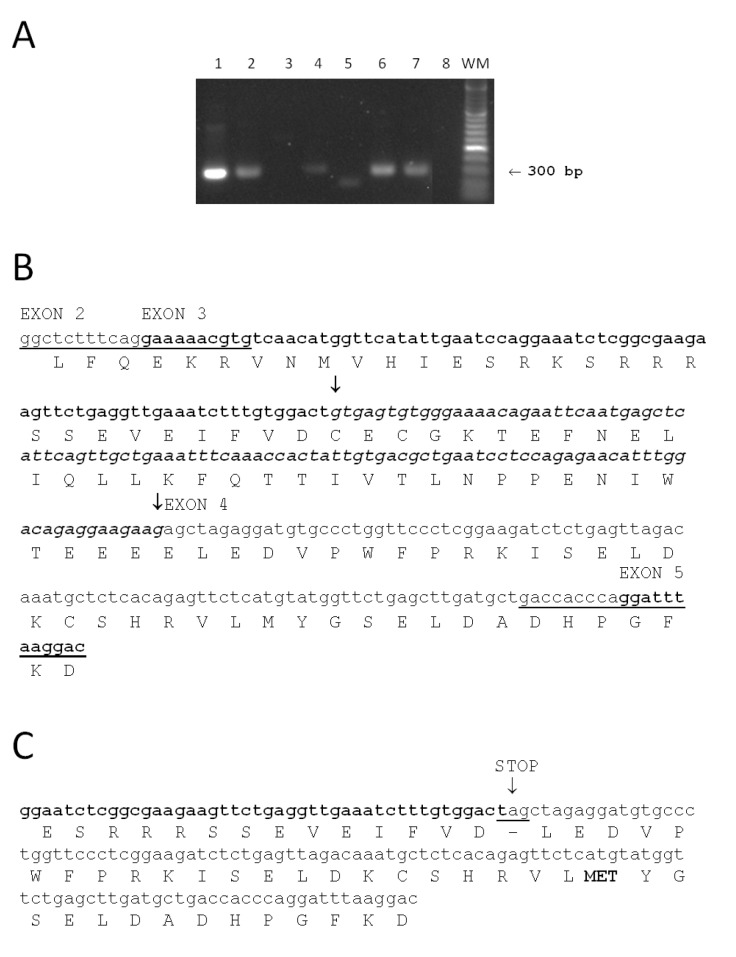
Analysis of TPH2 expression in human skin cells. (**A**) RT-PCR detection of TPH2 transcripts in human tissues and cell lines. 1—Brain, 2—skin, 3—immortalized (HaCaT) epidermal keratinocytes, 4—human dermal fibroblasts, 5—immortalized human melanocytes (PIG1 line), 6—primary human melanocytes (passage 4), 7—human adult ARPE19 retinal pigment epithelium cells line (passage 26), 8—control (no cDNA template), WM—molecular weight marker (100 bp DNA Ladder (O’Range Ruler, Fermentas)). Total RNA was isolated from skin biopsy or cell lines using a total RNA extraction kit, supplemented with RNAse-free DNAse Set (both Qiagen). Two micrograms of total RNA were used for reverse transcription with SuperScript First-Strand Synthesis System (Applied Biosystems, Foster City, CA, USA). Brain cDNA was purchased from Origene. Amplification of THP2 fragments was performed using specific set of primers MZ138 (GGCTCTTTCAGGAAAAACGTG) and MZ139 (GACCACCCAGGATTTAAGGAC) synthesized by Integrated DNA Technology Inc. (Coralville, IA, USA). The PCR products were separated by agarose gel electrophoresis and stained with ethidium bromide staining. Arrow shows the expected full length (300 bp) TPH2 fragment; (**B**) Nucleotide (small letters) and predicted amino acid (capital letters) sequences of the amplified full length TPH2 (300 bp) fragment. Sequences of primers MZ138 (GGCTCTTTCAGGAAAAACGTG) and MZ139 (GACCACCCAGGATTTAAGGAC) used for PCR amplifications are underlined (please note that for MZ139 reverse complementary sequence is shown). Exons (2, 3, 4 and 5) positions are indicated above nucleotide sequence and exons 3 and 5 are shown in bold. Spliced out mRNA fragment (italic) and corresponding protein sequence are labeled with arrows; (**C**) Nucleotide and predicted amino acid sequences of the short TPH2 (255 bp) transcript. This fragment was found only in immortalized (PIG1) human melanocytes. Fragment of exon 3 is shown in bold font, exon 4 is shown in normal font. Stop codon (underlined) introduced as a result of alternative splicing is labeled with an arrow. Alternative start of translation (codon MET) is shown in bold.

To complete our studies on serotonin and melatonin synthesis in the human skin, we examined human skin cells for expression of TPH2 (Figure 2). The expected full length 300 bp TPH2 fragment was found in brain, skin, primary dermal fibroblasts, and primary melanocytes, as well as in retinal pigment epithelium cell line ARPE19, but it was absent from HaCaT immortalized keratinocytes (Figure 2A). Interestingly, we detected a shorter TPH2 fragment in immortalized PIG1 human melanocytes (Figure 2B) representing an alternatively spliced variant. Sequence analysis showed the presence of stop codon introduced by alternative splicing that would prematurely terminate translation of TPH2 mRNA. In addition, the presence of an alternative start of translation (codon MET) may suggest production of an additional protein form of TPH2. These initial data warrant further investigation related to the expression of different forms of TPH1 and TPH2 in skin cells.

### 1.3. Melatonin Receptors in the Skin

Classical chronobiology identifies melatonin as the important regulator of the circadian and circannual bio-rhythms defined by its rhythmic night/day production and variations in the duration of the nocturnal melatonin peak [2,32,33]. In addition, melatonin can act as immunomodulator, modifier of endocrine activity, metabolism and a regulator of seasonal reproduction [34,35,36,37], while at the cellular and tissue levels it acts as a modulator of cell proliferation, differentiation, apoptosis with tumorostatic properties (reviewed in [25,38,39,40,41,42]). Local melatonin effects are related to its production in peripheral organs and accumulation in different body compartments including bile fluid, cerebrospinal fluid, gastrointestinal tract, bone marrow, ovary, eye, lymphocytes or skin (reviewed in [25,38,39,42,43]) and its differential distribution in the subcellular organelles [44,45,46]. Its effects are mediated by receptor dependent and independent mechanisms [17,39,47,48,49,50,51].

Melatonin receptors have been reported in the skin and skin cells [23,25,52,53,54,55,56,57,58]. These receptors include MT1 and MT2, with MT1 being the dominant receptor in the human skin [39,53,57]. The receptor MT1 is located in the following regions of the skin: *stratum granulosum*, *stratum spinosum*, upper and inner root sheath, eccrine sweat gland, and the endothelium of blood vessels, while MT2 is found only in the inner hair sheath, eccrine sweat glands, and the endothelium of blood vessels [23,54]. The expression of the MT receptor can also be affected by environmental factors [57]. For example, UV exposure promotes expression of both the MT1 gene and MT2 gene in melanocytes, epidermal keratinocytes, and melanocytes or it induces expression of alternatively spliced forms [57]. Although RORα [24,57,59,60] and RORγ [60] are expressed in the mammalian skin, none of them serves as the receptor for melatonin or its metabolites [39,60] although RORα was formerly considered a nuclear receptor for melatonin [39]. Thus, the search for putative nuclear receptor(s) for melatonin and its metabolites is still open for further research.

### 1.4. Overview of Melatonin Metabolism

There are three major melatonin metabolic pathways: classical, indolic, and kynuric [31,61,62]. The classical pathway for melatonin degradation starts with melatonin being metabolized by the CYPP450 family enzymes to 6-hydroxymelatonin [63]. The substrate 6-hydroxymelatonin (6(OH)M) then becomes more polarized by the addition of either sulfate or glucuronide [64].

In the indolic pathway, melatonin is metabolized by melatonin deacetylase to 5-methoxytryptamine (5-MTT) [65]. Monoamine oxidase-converts 5-methoxytryptamine to 5-methoxyindoleacetaldehyde [62]. 5-Methoxyindoleacetaldehyde is enzymatically metabolized to either 5-methoxyindole acetic acid (5-MIAA) by aldehyde dehydrogenase or 5-methoxytryptophol (5-MTOL) by alcohol dehydrogenase [62].

The kynuric pathway has two forms: enzymatic and nonenzymatic. Thus, melatonin is metabolized ezymatically by indoleamine 2,3 dioxygenase to produce *N*^1^-acetyl-*N*^2^-formyl-5-methoxykynuramine (AFMK) [66]. AFMK is then further metabolized by arylamine formamidase to become *N*^1^-acetyl-5-methoxykynuramine (AMK) [61]. AMK can react with carbamoyl phosphate, H_2_O_2_, and Cu^2+^ to form MQA (*N*[2-(6-methoxyquinazolin-4-yl] [67]. AMK can also (theoretically) react with either NO^+^, NO, or HNO to form AAMC (3-acetyamidomethyl-6-methoxycinnolinone) [68]. Melatonin has also been shown to go through the non-enzymatic kynuric pathway in skin cells using UV light [69].

Both indolic and kynuric pathways of melatonin metabolism are operative in the skin (Figure 1) [22,28,29,31,69]. The detectable metabolites of the pathway included 5-MTT, 5-MTOL [22,28,29], 2-hydroxymelatonin, AFMK and 6(OH)M [69] with 6(OH)M being the main metabolite in epidermal cells [28,31].

## 2. Melatonin as a “Guardian” of the Genome and Cellular and Tissue Integrity of the Skin

### 2.1. Skin as the Environmentally Most-Stressed Organ

Skin is exposed, both acutely and chronically, to a variety of physico-chemical factors, which are capable of producing a state of overwhelming oxidative stress if left unchecked [70]. To appropriately react to environmental stressors, the skin is armed with a sophisticated local neuro-endocrine-immune system [71] composed of classical neuropeptides and neuroendocrine regulators [72,73,74,75] including local melanocortin system [76], steroidogenic [28,77] and secosteroidogenic [78,79,80,81,82,83,84,85,86], neuroimmune [70] and metabolic [87,88] activities. A critical role in protection against ultraviolet (UV) wavelengths of solar lights is played by the melanin pigmentary system [89,90]. The melanin producing system can be regulated by a local melatoninergic system [25] and melanocortin system [76], which also protect the skin against UVR induced damage [23,69,70,91,92,93]; additional details related to this are summarized later in this report.

### 2.2. Melatonin as a Protectant against Oxidative Stress Imposed to the Skin

Since organisms are normally exposed to environmental insults in a circadian fashion, the prooxidant-antioxidant homeostasis in the skin is maintained through a complex network of cellular machinery and signaling events regulating oxidative stress and circadian rhythms. Accumulating evidence suggests that activities of antioxidant enzymes, such as, catalase, glutathione peroxidase, and superoxide dismutase oscillate rhythmically and in various species, glutathione peroxidase activity shows a circadian oscillation following the melatonin pattern [94,95]. A disrupted circadian clock may be a key initiating factor in skin diseases linked to mitochondrial damage associated with oxidative stress [96,97,98,99,100]. Mitochondria not only generate ROS/reactive nitrogen species (RNS) but are also the main target of their destructive actions leading to the accumulation of oxidative damage more rapidly than the rest of the cell. Mitochondrial proteins, DNA and thiols are considered the molecules most susceptible to oxidative modification.

Under oxidative stress conditions, melatonin and its metabolites likely operate as local antioxidants, attenuating mitochondrial damage. Indeed, melatonin protects proteins from free radical attack, both *in vivo* and *in vitro* and can, therefore, also be considered an anti-skin aging substance [23,52,101,102,103]; it also efficiently prevents the toxic effects of *tert*-butyl hydroperoxide (t-BuOOH) on brain and liver mitochondria by regenerating the reduced glutathione (GSH) content [104]. Melatonin’s major hepatic metabolite, 6-hydroxymelatonin, also significantly reduces KCN-induced superoxide anion generation in rat brain homogenates and inhibits low density lipoprotein oxidation [105,106], while NAS has displayed the pronounced anti-oxidative effects on ROS formation (either spontaneous or t-BuOOH-induced) in human peripheral blood lymphocytes [107]. NAS and 6-hydroxymelatonin, being more hydrophilic than melatonin, exert their free radical scavenging actions especially in the aqueous phase, or at the water-lipid interface. In contrast, melatonin positions itself within the lipid bilayer where it protects membrane proteins against free radical attack. Melatonin and its metabolites significantly attenuate oxidative protein modification in isolated rat liver mitochondria treated with Fe^3+^/ascorbate; these agents were more effective than GSH in this situation [83]. AFMK reduces lipid peroxidation, X-ray-induced oxidative protein and DNA damage [51,108]. In addition, it was shownthat melatonin in leukocytes has a higher reduction potential (0.73 V) than vitamin C (0.23 V) [109]. For instance, formation of highly toxic hydroxyl radicals occur in presence of certain concentrations of vitamin C, while to date melatonin has not demonstrated to exhibit pro-oxidant properties in normal cells. Regarding UV-induced ROS generation, which is tightly connected with photodamage, it was shown that melatonin is a stronger scavenger of free radicals compared to vitamin C or trolox, a vitamin E analog [110].

Emerging evidence suggests that melatonin may protect DNA against free radical damage not only by modulating the gene expression of antioxidant enzymes or scavenging hydroxyl radicals but also via regulation of several key genes involved in DNA damage repair pathways [47,111]. There is evidence that melatonin protects the cytochrome P450 system in the oxygen free radical generating system and confirms its role in the detoxication of mitochondrial H_2_O_2_ in the intermembranous space via interaction with oxoferryl cytochrome *c* [112,113]. In addition, mitochondrial P450-dependent metabolism of melatonin could mediate the activation of intermembranous Cu/Zn-superoxide dismutase via oxidative modification of its critical thiols by superoxide anion radical (O₂^•−^) [114]. Thus, some or much of the antioxidant activity of melatonin *in vivo* may be attributable to its metabolites, AFMK, NAS and 6-hydroxymelatonin, as formed in reactions catalyzed by microsomal or mitochondrial cytochrome P450 or cytochrome *c* [115]. It should be noted that all of the compounds are produced in the skin [27,31,59].

**Figure 3:**
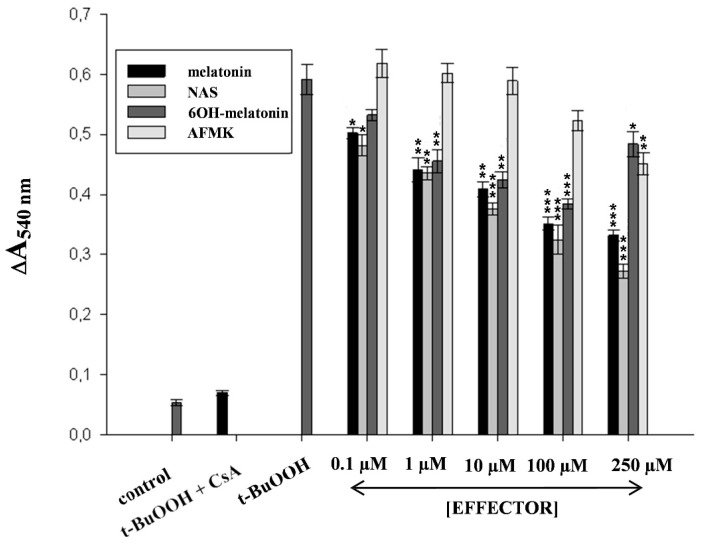
Effect of melatonin, 6-hydroxymelatonin, *N*-acetylserotonin and AFMK on t-BuOOH-induced swelling of rat liver mitochondria. Swelling was measured spectrophotometrically by monitoring the absorbance at 540 nm of suspensions of mitochondria, intact or treated with 500 µM tert-butyl hydroperoxide (t-BuOOH), with or without cyclosporin A (CsA) (5 μM), and in the presence or absence of effectors (0.1, 1, 10, 100, 250 µM). Mitochondria were isolated and treated as described previously [83,115]. Protective effect of melatonin, 6-hydroxymelatonin, *N*-acetylserotonin and AFMK (mitochondria treated by t-BuOOH in the presence of effectors) compared to corresponding samples without effectors was considered statistically significant and indicated as * *p* < 0.05; ** *p* < 0.01; *** *p* < 0.001 using the Student’s *t*-test. Data represent mean values obtained from 3 experiments.

The antioxidant potential of melatonin may also be decisive for its anti-apoptotic effect [116]. Increased mitochondrial formation of ROS triggers the mitochondrial pathway [112] of apoptosis by opening the transition pores due to oxidation of glutathione and specific thiol residues in the mitochondrial permeability transition pore (MPT) components, e.g., adenine nucleotide translocator [117]. Mitochondria permeabilization by oxidative stress has been widely studied as model for initiator of apoptosis. ROS generation and GSH oxidation caused by t-BuOOH initiate NADPH consumption and enhance mitochondrial Ca^2+^ uptake, leading to MPT and cell death [118,119]. Based on our findings, exposure of rat liver mitochondria to t-BuOOH results in progressive mitochondrial swelling (Figure 3). The swelling induced by the hydroperoxide is completely blocked by pre-incubation of mitochondria with CsA, a classical inhibitor of the MPT, confirming that swelling was caused by PTP opening. Melatonin, NAS, AFMK or 6-hydroxymelatonin did not induce mitochondrial swelling and, in fact, stabilized mitochondria exposed to the oxidant, and protecting them from t-BuOOH-induced swelling.

The protection afforded by melatonin against MPT induced by t-BuOOH may be related to indole’s ability to stimulate the activity of enzymes involved in the GSH-GSSG equilibrium by scavenging free radicals and thereby protecting critical thiols from oxidation. This is consistent with a report of melatonin reducing significantly mitochondrial ROS formation, as well as the PTP opening induced by t-BuOOH in mitochondria of rat brain astrocytes [120]. In UV-irradiated keratinocytes melatonin maintains mitochondrial membrane potential and inhibits intrinsic apoptosis pathways activated by mitochondrial-generated ROS [121]. Melatonin’s anti-apoptotic effects may involve, in addition to its potent besides antioxidant activities, additional mechanisms such as direct inhibition of the PTP [122].

Considering the findings mentioned above, melatonin likely plays a pivotal role in protecting cells (including skin cells) from death, by targeting its actions to a step before “the point of no return”. This anti-apoptotic activity of melatonin may be mediated, at least in part, by its metabolites (NAS and 6-hydroxymelatonin), which based on our results is just as efficient as the parent compound at suppressing the opening of the PTP induced by t-BuOOH (Figure 3). This is consistent with the observations that NAS is more effective than melatonin in protecting peripheral blood lymphocytes from death caused by t-BuOOH [107]. A possible explanation might be related to differences in indoleamine reactivity towards peroxyl and alkoxyl radicals generated from t-BuOOH in the presence of small amounts of transition metal ions or in the content of cytochrome *c* [123]. In at least one study, melatonin showed a lack of antioxidant activity against the peroxyl radical-induced lipid peroxidation in models of cell membranes and was less effective than NAS as a peroxyl radical scavenger in aqueous cell-free solutions [107]. Thus, NAS may scavenge peroxyl radicals more effectively than melatonin, especially when reducing damage caused by reactive species formed as a result of treatment with t-BuOOH. Furthermore, NAS was shown to prevent PTP opening induced by calcium, phosphate or neurotoxins [124], suggesting that it may prevent cell death, under, both, physiological or pathological conditions. It is important to emphasize that skin produces relatively high levels of NAS in an AANAT dependent and independent manner [9,10,19,20,27,29]. This capability indicates an important role for NAS in regulation of cutaneous stress responses, especially since it is metabolized in the skin to species other than melatonin, although these non-melatonin metabolites remain to be defined [10]. Furthermore, cutaneous NAS may have systemic activity after the entering circulation; this possibility awaits future studies.

Mitochondria do participate in the clearance of melatonin via conversion into NAS and 6-hydroxymelatonin; these are excreted as sulfate and glucuronide conjugates. However, mitochondria may also be a target for melatonin and its metabolites, since under physiological conditions, it increases the activity and expression of complexes I and IV of the electron transport chain (ETC). In addition, it restores their activities when previously reduced by pathological conditions [125,126,127]. Mitochondrial complexes that are major sites of ROS production and are also targets of their damaging effects. For example, ROS generated from ETC affect the activity of complexes I and IV via peroxidation of cardiolipin needed for their optimal function [128,129]. Complex IV is also inactivated by 4-hydroxy-2-nonenal, while NO or its derivatives (reactive nitrogen species) inhibit mitochondrial complex I [130]. Due to its antioxidant properties and localization in a superficial position of the lipid bilayers melatonin protects complexes I and IV from the destructive actions of free radicals and reactive species. Moreover, the redox potential of melatonin suggests that it could donate electrons to the ETC, thus improving mitochondrial respiration and increasing ATP [131]. 6-Hydroxymelatonin exhibits greater reducing potential for oxidized cytochrome *c* than melatonin itself. During mitochondrial respiration cyt *c* mediates electron shuttling between ubiquinol cytochrome *c* oxidoreductase (complex III) and cytochrome *c* oxidase (complex IV). Therefore, when an electron is removed from 6-hydroxymelatonin, it becomes available for donation by ferrocytochrome to cytochrome *c* oxidase, providing an additional contribution to mitochondrial energy production. Since electrons transferred by the respiratory chain are accepted by oxidized 6-hydroxymelatonin, this molecule would anchor a redox cycle that may effectively enhance electron flow through the ETC. Thus, hydroxylated melatonin may promote ATP synthesis, even more effectively than its parent compound.

Newly-formed 6-hydroxymelatonin can allow for electron transfer to occur in the terminal cytochrome *c* oxidase segment of the ETC, even in the presence of dysfunction in its initial steps; such an effect may be important in cases of deficient activity of mitochondrial complexes I, II and III (e.g., Parkinson’s disease) [132,133]. In addition, 6-hydroxymelatonin partially reverses the reduction of mitochondrial electron transport induced by KCN via complex IV inhibition, a cause of rapid and severe depletion of cellular ATP [105]. Melatonin also interacts with lipid bilayers and stabilizes mitochondrial inner membranes against oxidative stress, an effect that may also improve ETC activity [134]. Both, melatonin’s metabolites, NAS and 6-hydroxymelatonin have similar membrane stabilizing activity in a model of liver injury induced by alpha-naphthylisothiocyanate [135]. In the absence of oxidative stress, NAS and melatonin do not alter the physical properties of cellular membranes, while under stress they improve membrane fluidity [136,137]. It is presumed, therefore, that melatonin and NAS stabilize cellular membranes by preventing lipid peroxidation caused by free radicals [136,138]. Nevertheless, in contrast to NAS, melatonin and partly 6-hydroxymelatonin may position themselves in the membrane providing the lipid bilayer with local protection from free radical attack, and, thereby, maintaining optimal fluidity of cellular membranes. It is then possible that melatonin and/or its metabolites provide integrated effects on mitochondrial membrane stabilization, under different conditions (physiological or pathological). It must be emphasized here that 6-hydroxymelatonin is a major melatonin metabolite in the human skin [28,31]. Finally it has been hypothesized that melatonin may be synthesized in mitochondria [139]. If this is confirmed, it would obviously make melatonin, as well as its metabolites, readily available for protection of mitochondria from oxidative destruction.

### 2.3. Melatonin Protects Skin Cells against UVR

Since keratinocytes represent the major residential cell population in the epidermis (≥90% of epidermal cells), a majority of studies related to the protective effects of melatonin against UVR have used these cell populations [57,91,121,140,141,142]. The results of these studies are consistent with earlier findings on the protective effects of melatonin against UVB radiation in leukocytes [110,143]. Melatonin also suppressed starvation-induced apoptosis in HaCaT keratinocytes [53].

Our initial studies showed that pre-incubation with melatonin at 10^−4^ or 10^−3^ M concentrations was required for its ability to inhibit apoptosis and preserved cell viability of HaCaT keratinocytes exposed to 25 or 50 mJ/cm^2^ of UVB [140]. Our follow-up studies with the UVB dose of 50 mJ/cm^2^ not only confirmed that pharmacological doses of melatonin prevented apoptosis in keratinocytes but also showed that it attenuated the UVB-induced reductions in mitochondrial membrane potential [121]. These effects were followed by suppression of the activation of mitochondrial pathway-related initiator caspase 9 (casp-9), but not of death receptor-dependent caspase-8. Furthermore, melatonin down-regulated effector caspases (caspase-3/caspase-7) and reduced PARP activation. This prompted the conclusion that melatonin was active in UV-irradiated keratinocytes, maintains the mitochondrial membrane potential, inhibits the consecutive activation of the intrinsic apoptotic pathway all of which lead to a reduction of PARP activation, an indirect marker of DNA damage [121].

**Figure 4:**
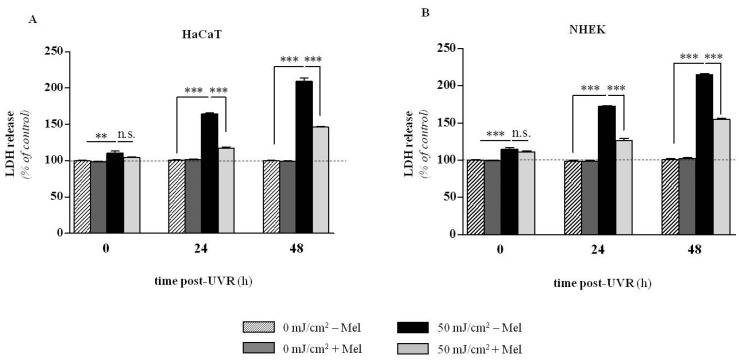
Presentation of UVR-induced release of LDH in human keratinocytes in dose- and time-dependent manner. Investigation was conducted using immortalized (HaCaT) (**A**) and normal (NHEK) (**B**) keratinocytes first pre-incubated with melatonin for 1 h (10^−3^ M) and irradiated with the UVB dose of 50 mJ/cm^2^. Data were presented as the mean ± SEM of three independent experiments. Values were normalized and expressed as percentage of the control value, *i.e.*, sham-irradiated sample (0 h 0 mJ/cm^2^) without melatonin (Mel). Statistically significant differences in melatonin *versus* non-melatonin treated samples at corresponding UVR doses and time points post-UVR were indicated as ** *p* < 0.01; *** *p* < 0.001; n.s., not significant, using the ANOVA with appropriate *post-hoc* testing (modified after Kleszczyński *et al.* [142] with permission from the publisher).

**Figure 5:**
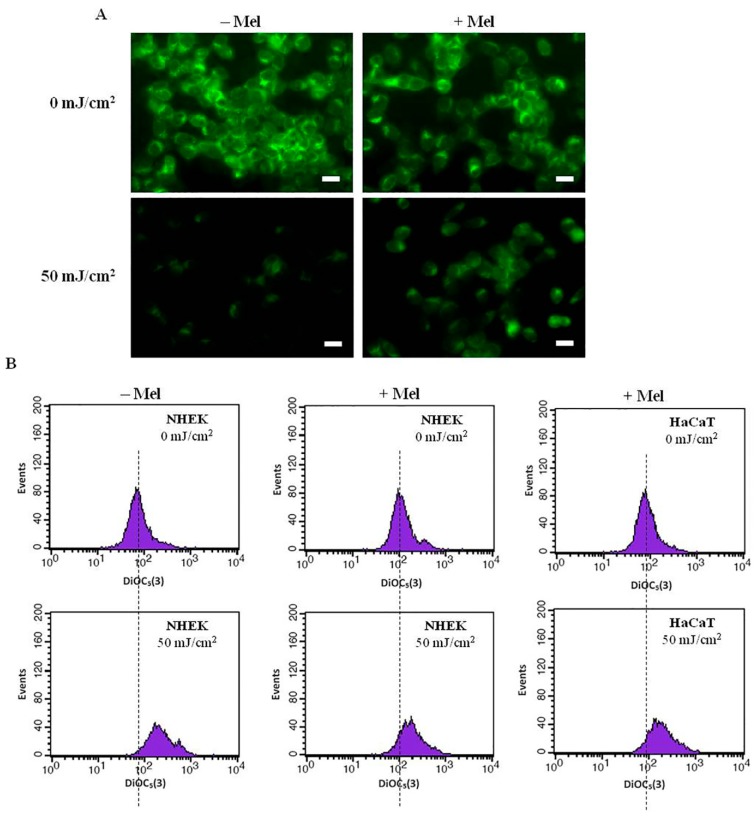
Protective effect of melatonin against UV-induced alterations within plasma membrane in human keratinocytes. (**A**) Fluorescent images of normal human keratinocytes (NHEK) (magnification, 40×) presenting the impact of UVR and melatonin. A representative experiment is shown. Bars = 20 μm; (**B**) Plasma membrane potential histograms obtained by flow cytometry after 24 h post-UVR (50 mJ/cm^2^) in HaCaT and NHEK keratinocytes. The horizontal axis indicates DiOC_5_(3) fluorescence intensity and the vertical axis indicates number of cells. The histograms shifted to the right upon UVR exposure (hyperpolarization of mbΔψ) while presence of melatonin reversed this effect (modified after Kleszczyński *et al.* [142] with permission from the publisher).

**Figure 6:**
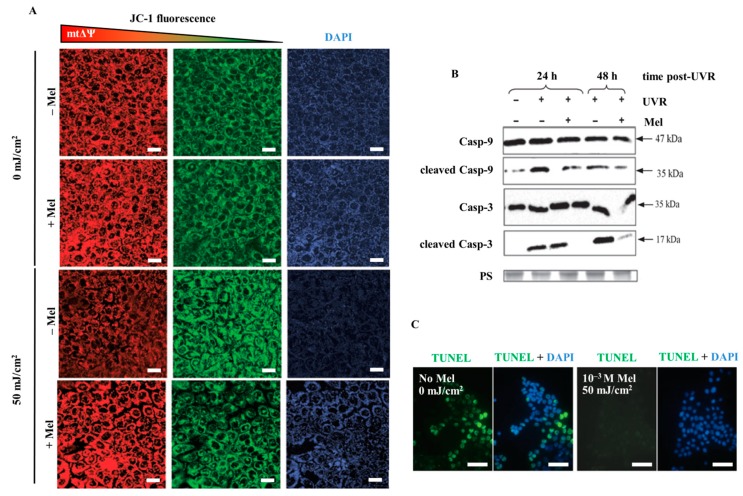
UVR-induced changes in mitochondrial transmembrane potential and protective action of melatonin (**A**) in HaCaT keratinocytes. Cells were pre-incubated with melatonin (10^−4^ M) and irradiated with the dose of 50 mJ/cm^2^. Mitochondrial membrane potential is indicated by JC-1 red fluorescence (**left** panels). Relative changes in mitochondrial membrane potential are expressed as shifts from red to green fluorescence (**middle** panels) and presented as the red to green ratio that produces blue fluorescence (**right** panel); (**B**) Subsequent analysis of activation of mitochondrial-dependent (intrinsic) activation of cascade of caspases 3 and 9 showed prominent cleavage of both proteins leading to increased number of apoptotic TUNEL positive cells (green) indicating on UVR-mediated DNA damage (**C**) Bars = 20 μm (magnification, 40×). Melatonin effectively protected the cells against these disturbances (modified after Fischer *et al.* [121] with permission from the publisher).

The most recent investigations document that melatonin significantly counteracts UVR-induced enhanced lactate dehydrogenase (LDH) release at the UVR dose of 50 mJ/cm^2^, both, in case of immortalized (HaCaT) and primary neonatal (NHEK) epidermal keratinocytes (Figure 4A,B) [142]. Additionally, melatonin is protective against UVB-induced decrease of pH_i_ [142] (Table 1, Figure 5A,B). Thus, we observed prominent disturbances within the plasma membrane potential (Figure 5A,B) and subsequent acidification of cytosol as a result of UV exposure (Table 1). It should be noted that these early stage alterations within the cells triggered the series of functional perturbations within intracellular organelles especially in mitochondria where dissipation of transmembrane mitochondrial potential (Figure 6A) occurred [121]. A similar protective effect of melatonin on preservation of mitochondrial membrane potential (ΔΨ) was shown in HaCaT keratinocytes treated with 1 mM H_2_O_2_ (data not shown). UVR-mediated uncoupling of oxidative phosphorylation led to activation of a cascade of caspases (casp-9 and casp-3) (Figure 6B) and which led to nuclear damage (Figure 6C) [121]. The addition of melatonin effectively reduced these effects. In this study melatonin also enhanced expression of anti-oxidative enzymes such as superoxide dismutase (SOD), catalase (CAT) and glutathione peroxidise (GPx) both at the RNA and protein levels [141,144].

**Table 1:** Calculated pH_i_ in human keratinocytes (HaCaT and NHEK). Cells were exposed to UVR and cultured post-UVR in time-dependent manner, stained with FDA, and analyzed for intracellular pH by flow cytometry. Data present pH_i_ mean ± SEM of three independent experiments (modified after Kleszczyński *et al.* [142] with permission from the publisher). Statistically significant differences compared to sham-irradiated cells at particular time end-points post-UVR without melatonin are presented as follows: ^#^ *p* < 0.001. Protective effect of melatonin compared to corresponding samples without melatonin was considered statistically significant and indicated as ^+^ *p* < 0.05, ^++^ *p* < 0.01, ^+++^ *p <* 0.001.

Time Post-UVR (h)	Intracellular pH (pH_i_)			
	0 mJ/cm^2^		50 mJ/cm^2^	
	–Mel	+Mel	–Mel	+Mel
**HaCaT**				
0	7.40 ± 0.01	7.40 ± 0.02	7.22 ± 0.03 ^#^	7.22 ± 0.05
24	7.37 ± 0.03	7.39 ± 0.02	6.40 ± 0.04 ^#^	6.56 ± 0.01 ^+^
48	7.31 ± 0.05	7.33 ± 0.05	6.08 ± 0.02 ^#^	6.33 ± 0.03 ^+++^
**NHEK**				
0	7.40 ± 0.03	7.40 ± 0.02	7.14 ± 0.05	7.16 ± 0.04
24	7.36 ± 0.03	7.39 ± 0.03	6.11 ± 0.02 ^#^	6.43 ± 0.02 ^++^
48	7.34 ± 0.04	7.37 ± 0.03	5.93 ± 0.05 ^#^	6.28 ± 0.02 ^+++^

**Figure 7:**
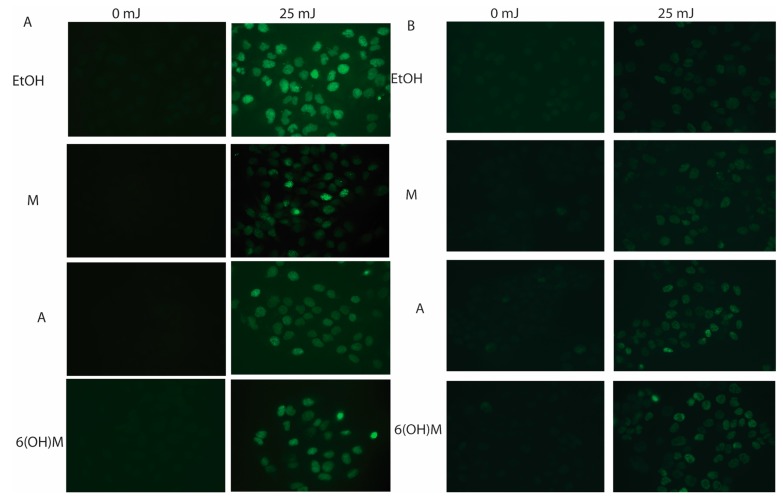
Melatonin (M), 6-hydroxymelatonin (6(OH)M) and AFMK (A) treated keratinocytes decrease CPD formation (**A**) or increase of p53 phosphorylated at Serine 15 (**B**) after UVB exposure. HEKn keratinocytes were treated with melatonin or its derivatives for 24 h before UVB exposure. Cells were exposed to UVB intensities of 25 mJ/cm^2^ and immediately treated again with melatonin or its derivatives for 3 h (**A**) or 12 h (**B**). Cells were fixed and stained with anti-CPD (**A**) or anti-phosphorylated p53S15 (**B**) antibodies (green) as described in [141].

Most recently, we investigated the protective actions of melatonin and its metabolites: 6-hydroxymelatonin, AFMK, NAS, and 5-MT in human keratinocytes against a range of doses (25, 50, and 75 mJ/cm^2^) of UVB [141]. NAS, melatonin and its metabolites caused significant reduction in the generation of ROS in normal and immortalized epidermal keratinocytes exposed to UVB. Also, each of these agents limited the nitrite and H_2_O_2_ levels that were induced by UVB, enhanced levels of reduced glutathione in keratinocytes and preserved viability of UVB-irradiated keratinocytes in a dose-dependent manner [141]. We note here an excellent study by Sarti *et al.* [145] showing crosstalk between melatonin and NO via mitochondria in HaCaT cells. In addition, NAS, melatonin and its derivatives enhanced the DNA repair capacity of UVB-induced pyrimidine photoproducts (6-4) or cyclobutane pyrimidine dimers (CPD) generation in human keratinocytes (Figure 7A). Finally, these compounds elevated the expression of p53 phosphorylated at Ser-15 but not at Ser-46 or its non-phosphorylated form as a part of protective response against UVB (Figure 7B). On the basis of these data we concluded that melatonin, its precursor NAS, and its metabolites 6-hydroxymelatonin, AFMK, 5-MT, all of which are endogenously produced in keratinocytes, protect these cells against UVB-induced oxidative stress and DNA damage [141]. It was also noted that for UV-exposed fibroblasts, only 56% of the cells survived upon UV exposure (140 mJ/cm^2^), while cells pre-incubated with 10−9 M melatonin caused a cell survival rate of 92.5% which was paralleled by significant reduction of lipid peroxidation and cell death [146]. In addition, comparative experiments using UV-treated fibroblasts showed a similar correlation in cell viability in presence of 10−7 M melatonin [147]. Finally, the most recent study suggested that melatonin at 10−3 M can protect NIH3T3 fibroblasts against UVA irradiation (15 J/cm^2^) [148]. The above studies indicate that melatonin can also protect dermal fibroblasts against damage induced by UVB and UVA exposure. The protective effect against UVA remains to be confirmed in human dermal fibroblasts.

### 2.4. Melatonin Protects against Skin Photodamage

**Figure 8:**
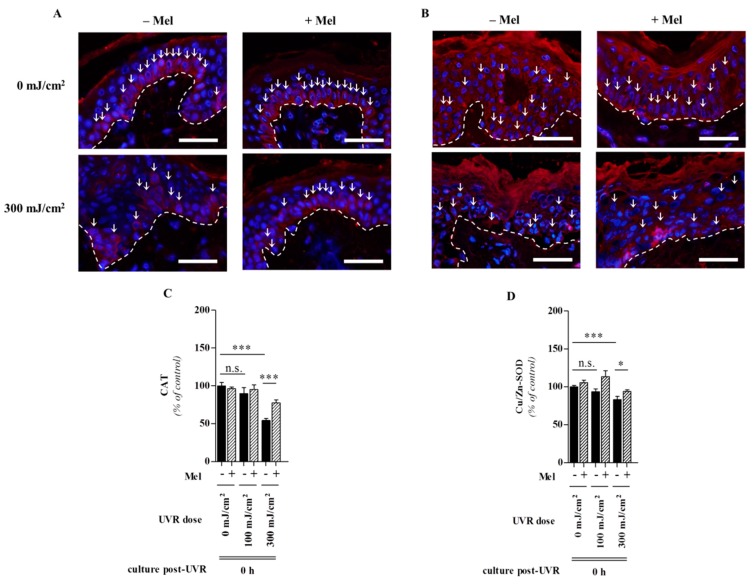
Protective effect of melatonin against UVR-mediated decrease of anti-oxidative enzymes in human skin in dose-dependent manner. UVR-induced decrease of CAT (**A**) or Cu/Zn-SOD (**B**) *in situ* protein expression was noticed directly post-UVR at the dose of 300 mJ/cm^2^ and melatonin induced enhanced antioxidant enzyme expression. Enzymes were detected using antibodies conjugated with rhodamine (red), DAPI was used for the nucleus (blue). One representative experiment of three is shown. Dashed line shows the basement membrane. Arrows show CAT and Cu/Zn-SOD positive cells. Bars = 50 μm (magnification, 500×). Evaluated data, (**C**,**D**), were presented as pooled means ± SEM of three independent experiments containing six images taken per condition. Values were expressed as percentage of the control value, *i.e.*, sham-irradiated without melatonin at 0 h post-UVR. Statistically significant differences were indicated as * *p* < 0.05; *** *p* < 0.001; n.s., not significant, using the ANOVA with appropriate *post hoc* testing (modified after Fischer *et al.* [144] with permission from the publisher).

**Figure 9:**
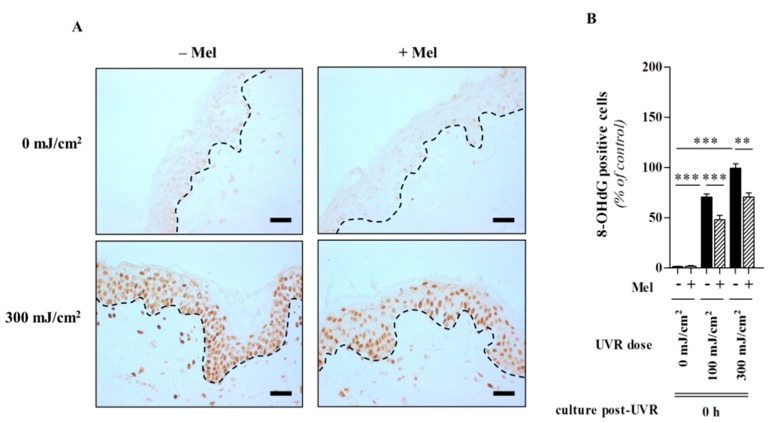
Melatonin significantly decreases the dynamics of formation of UVR-induced oxidative DNA damage, namely 8-hydroxy-2'-deoxyguanosine in human skin. Sections were labeled using immunohistochemical staining (**A**) for 8-OHdG and were detected by catalyzed signal amplification using 3,3'-diaminobenzidine (yields brown-colored precipitate). One representative experiment of three is shown. Dashed line shows the basement membrane. Bars = 50 μm (magnification, 200×); Evaluated data (**B**) were presented as pooled means ± SEM of three independent experiments containing six taken images per condition. Statistically significant differences were indicated as ** *p* < 0.01; *** *p* < 0.001; n.s., not significant, using the ANOVA with appropriate *post hoc* testing, (modified after Fischer *et al.* [144] with permission from the publisher).

Since free radicals and oxidative damage are in fact main factors in intrinsic and UV-induced skin aging [149] and given that the melatonin attenuates free radical damage, the actions of this indole represent an important role in melatonin-mediated protection against UV solar skin damage and skin aging [23,52]. This key observation makes melatonin a promising candidate in terms of a potent antioxidant and protective substance in skin photobiology [23,150]. By identifying that UV-induced melatonin metabolism leads to generation of melatonin-derived antioxidant metabolites in human keratinocytes where the functions as strong anti-oxidative agents, several molecules obviously contribute to the melatoninergic anti-oxidative system (MAS) of the skin [69]; hence, the cutaneous serotoninergic/melatoninergic system has secured a place under the sun [23]. The MAS functions in the skin as an important barrier against UV-induced oxidative stress-mediated damaging events on DNA, lipid, protein and preserves cellular integrity. Since all metabolites are lipophilic, they would diffuse into every skin compartment, thus extending the MAS beyond the epidermis. During all steps of this process, ROS are scavenged, and resulting damaging events are either indirectly or directly abrogated due to a reduction in lipid peroxidation, protein oxidation, and mitochondrial damage and DNA destruction. This concept is further supported by protective actions of not only melatonin but also of its precursor NAS and melatonin’s metabolites against UVB-induced oxidative stress and DNA damage [141]. This makes the melatoninergic anti-oxidative cascade highly potent in reducing the molecular damage resulting from the extensive amounts of free radicals that are generated under UV solar radiation. These observations suggest melatonin as a very promising agent to protect the skin against this major environmental stressor and causative factor of skin aging and tumour initiation and promotion. Our recent investigations showed that full-thickness human skin pre-incubated for 1 h with melatonin (10^−3^ M) was significantly protected from harmful effects of UVR. We also reported that UV-exposed skin was associated with a prominent decrease of CAT and Cu/Zn-SOD within the epidermis directly post-UVR at the dose of 300 mJ/cm^2^ (Figure 8A,B) [144]. Presence of melatonin significantly reversed these changes. In these studies, oxidative DNA damage was elevated by measuring the formation of the DNA base-oxidized intermediate, 8-hydroxy-2'-deoxyguanosine (8-OHdG) (Figure 9A,B) [144]. These *ex vivo* studies on human skin provide strong evidence for an *in vivo* protective role of melatonin against UVR-induced skin damage.

## 3. Conclusions

Since the discovery of the strong antioxidant properties of melatonin [151], a tremendously wide spectrum of targets and effects of melatonin in human and animal biology has evolved, thus showing that melatonin is an important bioregulator as well as pluripotent and essential protective agent in many cells, tissues and compartments of unicells, animals and humans [152,153]. Within this framework, the human skin is not only a target for the protective actions of melatonin but also a site of a melatonin synthesis and metabolism [23,25,31]; this latter event indicates the important role for its metabolites in protection against the UVR induced damage. While melatonin exerts many effects on cell physiology and tissue homeostasis via membrane bound melatonin receptors [39,49,50] the strong protective effects of melatonin against the UVR-induced skin damage seen at its high (pharmocological) concentrations indicate that these are mainly mediated through its potent and diverse direct radical scavenging actions as well as to its metabolic and anti-oxidative enzyme stimulatory effects [25,91]. Finally, its ability to promote the DNA repair system or activation of its ability to protect against DNA damage by stimulating p53 is considered important [141]. Some of these could also be mediated by putative “melatonin nuclear receptors”, which remain to be definitely defined.

The destructive effects of the main environmental skin stressor, UVR, are significantly counteracted or modulated by melatonin in the context of a complex intracutaneous melatoninergic anti-oxidative system with UVR-enhanced or UVR-independent melatonin metabolites, such as AFMK, possibly being more important in this context than melatonin itself [23,69]. Therefore, endogenous intracutaneous melatonin production, together with topically-applied exogenous melatonin or metabolites would be expected to represent one of the most potent anti-oxidative defense systems against the UV-induced solar damage to the skin [23,25,52,91]. In summary, a central question is whether melatonin can be exploited therapeutically as a protective agent, as a general “skin survival factor” with anti-genotoxic properties or as a “guardian” of the genome and cellular integrity with clinical applications in UVR-induced pathology that include cancerogenesis and skin aging.
